# Calorimetric Properties of Some Alkali Pentaborate Hydrates From 15 to 370 °K

**DOI:** 10.6028/jres.068A.037

**Published:** 1964-08-01

**Authors:** George T. Furukawa, Martin L. Reilly, Jeanette H. Piccirelli

## Abstract

Measurements of the heat capacity of ammonium pentaborate tetrahydrate (NH_4_B_5_O_8_·4H_2_O), potassium pentaborate tetrahydrate (KB_5_O_8_·4H_2_O), and sodium pentaborate pentahydrate (NaB_5_O_8_·5H_2_O) were made in the range of about 15 to 370 °K and the data were used to obtain a table of smoothed values of thermodynamic functions from 0 to 370 °K. The measurements on sodium pentaborate pentahydrate were terminated at 345 °K because the temperature drifts that were observed above this temperature were considered to arise from gradual volatilization of the water of hydration.

## 1. Introduction

As a part of the program at the National Bureau of Standards to provide thermodynamic data on boron compounds, measurements of the heat capacity have been made on ammonium pentaborate tetrahydrate (NH_4_B_5_O_8_·4H_2_O), potassium pentaborate tetrahydrate (KB_5_O_8_·4H_2_O), and sodium pentaborate pentahydrate (NaB_5_O_8_·5H_2_O). (Henceforth, the abbreviations APT, PPT, and SPP will be used synonymously with the three respective alkali pentaborate hydrates.) These substances have the highest percentage of boric oxide (B_2_O_3_) content of the commonly available hydrated borates. The data were used to obtain smoothed values of heat capacity, enthalpy, enthalpy function, entropy, Gibbs free energy, and Gibbs free energy function from 0 to 370 °K.

The hydrates of the alkali pentaborates investigated would be more properly formulated as (NH_4_)H_4_B_5_O_10_·2H_2_O, KH_4_B_5_O_10_·2H_2_O, and NaH_4_B_5_O_10_·3H_2_O. The “hydrated” pentaborate ion, H_4_B_5_O_10_^−^, consists of two six-atom rings lying in perpendicular planes joined by a common tetrahedrally coordinated boron atom [1].^1^ Each of the four trigonal boron atoms is attached to two oxygen atoms in the ring and to a hydroxyl group

**Figure:**
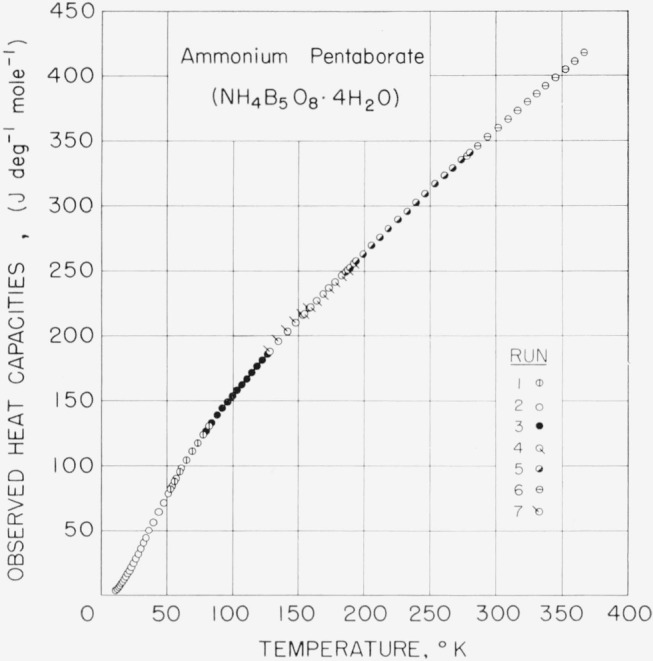

The water of hydration (two each in ammonium and potassium and three in sodium pentaborate) seems to be associated in some way with the oxygen atoms of the tetrahedral boron [2, 3]. The dihydrate of the sodium compound has not been isolated [3]. The trihydrate of lithium pentaborate and the dihydrates of rubidium and cesium pentaborates have been observed [3]. The anhydrous compound KB_5_O_8_ is known [4] but the anhydrate of APT and SPP has not been isolated [3]. The thermodynamics of these and other hydrated polyborates should be of interest for comparison with hydrated polysilicates, polyphosphates, and other structurally related substances.

## 2. Apparatus and Method

The heat-capacity measurements were made in an adiabatic calorimeter similar in design to that described previously [5]. The sample container was suspended within the adiabatic shield system by means of a nylon string instead of the filling tube shown in the above reference. Details of the calorimeter used and its operation will be described in a subsequent publication.

**Figure 1:**
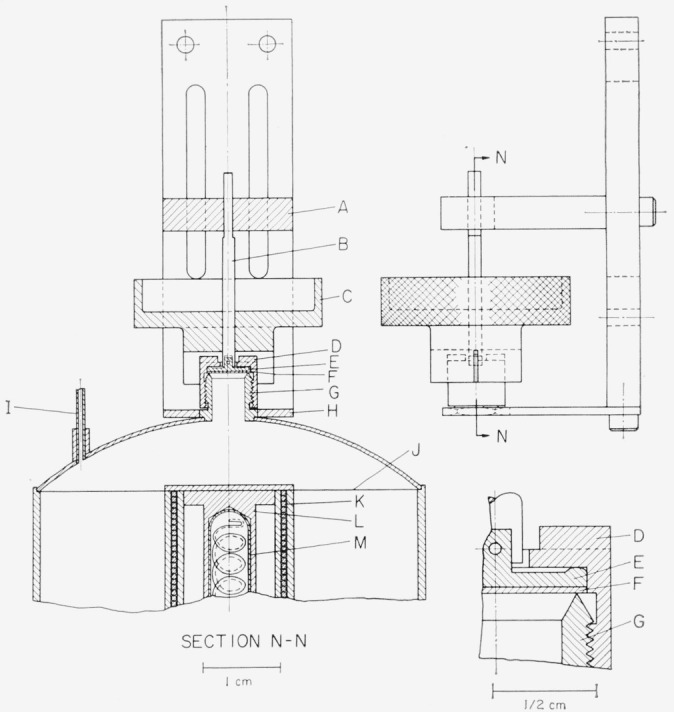
Screw-cap seal and sealing assembly for the sample container AAdjustable arrest with slot to keep rod B from turning.CWrench for turning screw-cap D.EMushroom-shaped plate that presses the gold gasket F against the sealing edge of the threaded tube G. Rod B prevents E from turning.HWrench held during the sealing process against a wrench flat at the base of G.ITinned copper tube for testing the screw-cap seal for vacuum and for the final sealing.JTop edge of radially arranged, tinned copper vanes.KHeater wire for the calorimeter vessel.LCopper case for the platinum thermometer case M.

Briefly, the sample was sealed in a copper container of about 125 cm^3^ capacity. The method for filling and the subsequent sealing of the container is shown schematically in figure 1. The sample was poured through the ¼ in. opening in the threaded member G, which was later sealed by means of a 0.01 in. thick gold disk F and the accessory supporting components D and E. During the sealing process, the mushroom-shaped member E was held securely from turning by means of A and B so that the gold disk F would be pressed tightly, without turning, against the sealing edge of G. The polished ridge on E decreased the “turning” friction between D and E. The screw-cap D was tightened against E by turning the knurled knob of wrench C. When the container was sealed, the sealing assembly (A, B, C, and H) was removed. Previous tests on simulated systems have shown that the seal was vacuum tight under the conditions of temperature cycling in the temperature range of the measurements and that the gold disk could be used three or four times or more without leakage. In addition a helium-gas leak detector was used to test the screw-cap seal with each sample through the auxiliary tube I.

The final seal was made by pinching and cutting the 116 in. copper tubing I which was previously tinned on both inner and outer surfaces. The pinching was done over about ½ in. of the tubing and a hot soldering tool was applied along the pinched portion of the tubing so that the tin on the inner surface would form a tight seal before the cutting was done at the pinched portion. Additional solder was applied at the cut edge as an added precaution against leakage.

In order to attain a rapid temperature equilibrium, tinned copper vanes were arranged radially from the central well to the outer wall of the container and held in place by a thin coating of pure tin applied to the inner surfaces. The radially arranged vanes were terminated in the plane indicated by J in figure 1 to permit easy distribution of sample when poured through the opening in G. A small quantity of helium gas was also sealed in with the sample to facilitate temperature equilibrium. The central well contained a heater-platinum resistance thermometer assembly (shown as K, L, and M in fig. 1).

The outer surface of the container and the adjacent inner surface of the adiabatic shield, within which the container was suspended by means of a nylon string, were gold plated and polished to minimize radiative heat transfer. The space around the container and shield was evacuated to a pressure of 10^−5^ torr or less (1 torr = 1/760 atm = 1 mm Hg) to make negligible the heat transfer by gaseous conduction and convection. During the heat-capacity experiments the temperature of the shield was maintained as close as possible to that of the container surface by means of shield heaters and constantan-Chromel-P differential thermocouples. Two sets of thermocouples, one of three junctions and the other of two, and three individual heaters were used in the control of the adiabatic shield and lead-wire temperatures.

The electrical power input was measured by means of a Wenner potentiometer in conjunction with a standard cell, volt box, and standard resistor. The volt box was assembled from two standard resistors, 100 and 10,000 ohms, the voltage being measured across the 100-ohm resistor. Since this is a relatively low-resistance voltage box, the resistance of the potential leads to the calorimeter heater was determined as a function of temperature. Over the temperature range of measurements, the volt-box “factor” changed up to 2 to 3 parts in 10,000 because of the change in the resistance of the potential leads with temperature. The volt-box factor was determined to better than 1 part in 10^5^.

The time interval of heating was measured by means of a precision timer operated on a 60 Hz frequency based on a 100 kHz quartz oscillator maintained at the National Bureau of Standards. The oscillator is stable to 0.5 ppm. The timer was compared periodically with seconds signals based also on the 100 kHz quartz oscillator. The timer deviations were never greater than 0.02 sec per heating period, which was never less than 2 min.

Temperatures were measured by means of a platinum-resistance thermometer and a high-precision Mueller bridge. The thermometer was calibrated by the Temperature Physics Section of the NBS. The calibration above 90 °K was in accordance with the 1948 International Practical Temperature Scale [6], and between 10 and 90 °K in accordance with the NBS–1955 provisional scale, which is maintained by a set of platinum-resistance thermometers that had been compared with a helium-gas thermometer.

At the Tenth General Conference held in 1954, the General Conference on Weights and Measures adopted a new definition of the thermodynamic temperature scale by assigning the temperature 273.16 °K to the triple-point temperature of water [6]. The provisional temperature scale as it is presently maintained at the National Bureau of Standards, and referred to as degrees K (NBS–1955), is numerically 0.01 deg lower than the former NBS–1939 scale [7]. The observed temperatures given in this paper conform with these new definitions of the temperature scales. The temperatures in degrees Kelvin above 90 °K were obtained by adding 273.15 deg to the temperatures in degrees Celsius (International Practical Temperature Scale [6]).

The 1961 atomic weights based on C^12^ were used to convert the mass of samples investigated to molal basis [8].

## 3. Analysis of Experimental Measurements

The measurements of heat capacity were made in the range of about 15 to 370 °K. Two sets of measurements were made, one on the container filled with sample and the other on the empty container. The usual precaution was observed to maintain the temperature increment of heating sufficiently small to minimize the correction for curvature of the heat-capacity function. The curvature correction was made wherever significant according to the procedure previously described [9].

After making the curvature corrections for the two sets of measurements, the heat-capacity values of the empty container were plotted on a large scale as deviations from approximate empirical equations. Smoothed values of the heat capacity at equally spaced integral temperatures were then obtained by combining the smooth deviation curves and the empirical equations. The temperature ranges of the empirical equations were overlapped and the values that joined most smoothly were selected. The smoothness of the tabular values was checked by examining the smoothness of the third and fourth differences. Wherever necessary a numerical smoothing process was employed [10].

The net heat capacities (heat capacity of the sample) were obtained by subtracting the heat capacity of the empty container from that of the container plus sample at corresponding temperatures. The values of heat capacity of the empty container were obtained by interpolation in the smoothed table described above. The net heat capacities were corrected for any differences in the mass of the container in the two sets of measurements. Corrections were made also wherever significant for the heat capacity of helium gas in the container. The net values of the heat capacity were then finally converted to molal basis [8] which are referred to in the following sections of this paper as “observed values of the heat capacity.” The heat capacity of the samples in these measurements was 80 ± 3 percent of the “gross” over the entire range of the measurements.

Smoothed values of the heat capacity of each substance were then obtained at equally-spaced integral temperatures by plotting on a large scale the deviations of the observed values from empirical equations and following the procedures similar to those previously outlined for the measurements on the empty container. Debye heat capacity functions, fitted to the experimental values at the lower temperatures, were used for extrapolation to 0 °K.

The thermodynamic properties for each substance were derived from the smoothed values of the heat capacity by procedures previously described [11].

## 4. Samples

The pentaborate samples obtained from the Pacific Coast Borax Company were in the form of fine crystals. Chemical analyses supplied with the sample are given in tables 1, 2, and 3. Analyses for B_2_O_3_, alkali oxide, and water were independently made on the samples by R. A. Paulson of the Applied Analytical Research Section of the Bureau. These results are summarized also in tables 1, 2, and 3 for comparison. The two sets of analyses are in fair agreement.

The ammonia in APT was analyzed by distilling the ammonia from a sample placed in a Kjeldahl apparatus and titrating with 0.1 *N* hydrochloric acid solution. The hydrochloric acid solution was standardized with single-crystal ammonium dihydrogen phosphate from which the ammonia was distilled from the Kjeldahl apparatus in the same manner as the APT sample.

The sodium and potassium in the samples were analyzed gravimetrically. The boron in the respective pentaborate was removed by evaporating to dryness six times with hydrochloric acid and methyl alcohol. The borate is removed in the process as volatile methylborate. The NaC*l* or the KC*l* formed was finally ignited at 700 °C and weighed.

The boron was analyzed as boric acid. A sample was dissolved in water and the *p*H adjusted to 7.0. Mannitol was added and the boric acid titrated with 0.1 *N* NaOH solution which had been standardized with pure boric acid.

The water of hydration was determined by heating a sample in a muffle furnace at 450 °C until a constant weight was obtained. The loss of weight of the ammonium compound at the above temperature was more than the expected amount of water. An additional analysis made on the substance in a tube furnace with a stream of dry argon also showed excessive loss of mass. Ievin’sh et al. [12] found that the last trace of water of hydration was not removed in APT until about 250 °C and that ammonia began to vaporize from about 140 °C. No determination of the water was, therefore, obtained on APT.

The analyses on PPT and SPP were normalized to 100 percent shown in the last column of tables 2 and 3, respectively. The low (NH_4_)_2_O and B_2_O_3_ content in APT suggests that the impurity is B(OH)_3_. Similarly, in PPT the low B_2_O_3_ and high H_2_O content with almost the theoretical content of K_2_O suggest that the impurity is B(OH)_3_. (The B_2_O_3_ content is lower and H_2_O content higher in B(OH)_3_ than in APT, PPT, or SPP.) The high Na_2_O, low B_2_O_3_, and high H_2_O content in the SPP sample indicate that the impurity is probably Na_2_B_4_O_7_·10H_2_O (borax). (Borax has a higher Na_2_O, lower B_2_O_3_, and higher H_2_O content than SPP.) The percentages of the suspected impurities calculated on the bases of the alkali oxide, boric oxide, and water contents obtained in the chemical analyses are summarized in table 4.

Because of the closeness of the B_2_O_3_ content of B(OH)_3_ to that of PPT, the error in the analysis of B_2_O_3_ would indicate directly the uncertainty in the content of B(OH)_3_ impurity in PPT. The comparison of the literature values (range: 15 to 300 °K) of the heat capacity of B(OH)_3_ [13] with the observed values of the PPT sample showed that the heat capacity of B(OH)_3_ is at most about 17 percent higher than PPT on the basis of mass. Considering also the uncertainty in the analysis of PPT for B_2_O_3_, the PPT sample was taken to be 100 percent pure in analyzing the experimental data.

The comparison of the observed heat capacity of the APT sample with that of B(OH)_3_ [13] showed that the heat capacity of the two materials differs generally within ±2 percent on the basis of mass. Therefore, the APT sample was also considered 100 percent pure in the analysis of the experimental data

No heat-capacity data on borax were found in the literature. The heat capacity of SPP and borax was assumed the same on the basis of mass.

## 5. Results

### 5.1. Ammonium Pentaborate Tetrahydrate, NH_4_B_5_O_8_·4H_2_O

**Figure 2:**
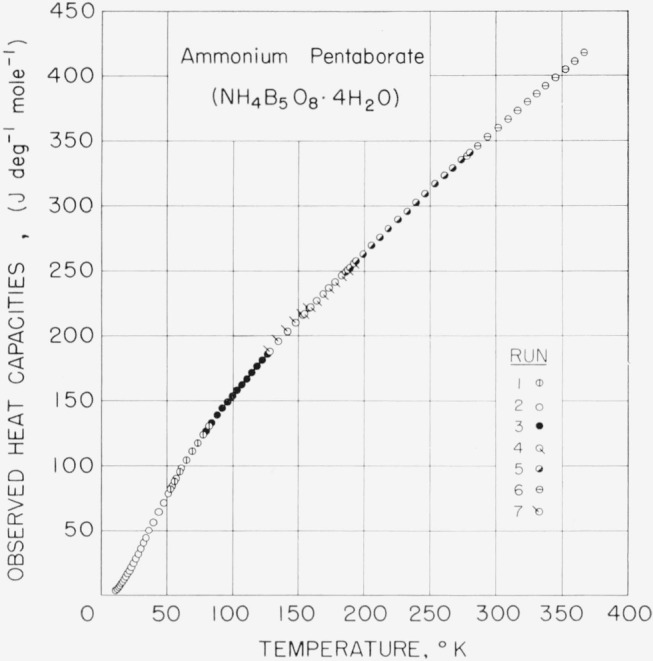
Observed heat capacities of ammonium pentaborate tetrahydrate, *NH_4_B_5_O_8_·4H_2_O.*

A 126.557 g sample of APT was investigated in the range 11 to 370 °K. The observed values of molal heat capacity are given in table 5, and plotted in figure 2. Values of molal heat capacity and derived thermodynamic functions were obtained at equally spaced integral temperatures. These are listed in table 6.

### 5.2. Potassium Pentaborate Tetrahydrate, KB_5_O_8_·4H_2_O

**Figure 3:**
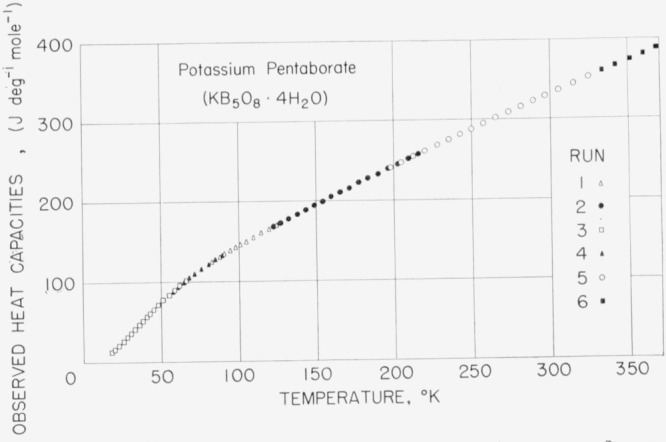
Observed heat capacities of potassium pentaborate tetrahydrate, *KB_5_O_8_·4H_2_O.*

A 141.366 g sample of PPT was investigated from about 17 to 370 °K. The observed values of molal heat capacity are listed in table 7 and plotted in figure 3 to show the general shape of the heat-capacity curve. Smoothed values of the heat capacity obtained from the experimental data and derived thermodynamic functions are listed in table 8.

### 5.3. Sodium Pentaborate Pentahydrate, NaB_5_O_8_·5H_2_O

**Figure 4:**
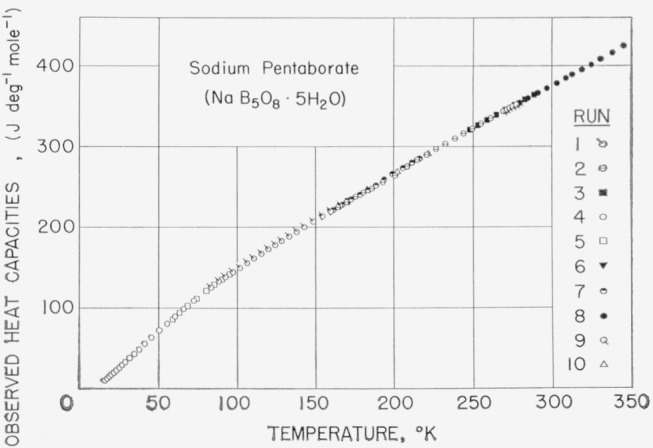
Observed heat capacities of sodium pentaborate pentahydrate, *NaB_5_O_8_·5H_2_O.*

A 177.320 g sample of SPP was investigated. Downward temperature drifts were observed in the measurements above 345 °K. Blasdale and Slansky [14] reported that SPP could be heated in an open container up to 70 °C without appreciable loss in weight, but when heated to 116 °C it formed a viscous liquid and began to lose water. On the bases of the observations of Blasdale and Slansky and of the high sensitivity of the calorimeter to any heat effects (0.0001 W or smaller), it seems likely that the downward temperature drifts observed are due to gradual dehydration of the SPP sample. Therefore, the data above 345 °K are considered inaccurate and are not reported. The observed molal values of heat capacity are given in table 9 and plotted in figure 4. The derived thermodynamic properties are listed in table 10 from 0 to 345 °K.

## 6. Discussion

In a series of papers Staveley et al. [15, 16, 17] investigated the contribution of the torsional or rotational motion of the ammonium ion to the heat capacity of ammonium salts with large symmetrical anions. By investigating the heat capacity of the ammonium and the corresponding isomorphous potassium and rubidium salts the heat-capacity contribution from the torsional oscillation or rotation of the ammonium ion was estimated by subtraction, assuming that the heat-capacity contributions from Cp-Cv, internal and torsional motions of the anion, and the lattice vibrations were the same in the two salts. (Hereafter the torsional or rotational heat capacity contribution of the NH_4_^+^ ion will be designated ∆*C_τ_*(NH_4_^+^).) The small contribution from the internal motions of the NH_4_^+^ ion was calculated using the assigned frequencies of Wagner and Hornig [18]. If the residual heat capacity obtained had a limiting value of $\frac{3}{2}R$ or 3*R*, a free rotation or a classical torsional oscillation, respectively, was suggested. For restricted rotator behavior a rise to a maximum followed by a decrease to a limiting value with increasing temperature is to be generally expected.

**Figure 5:**
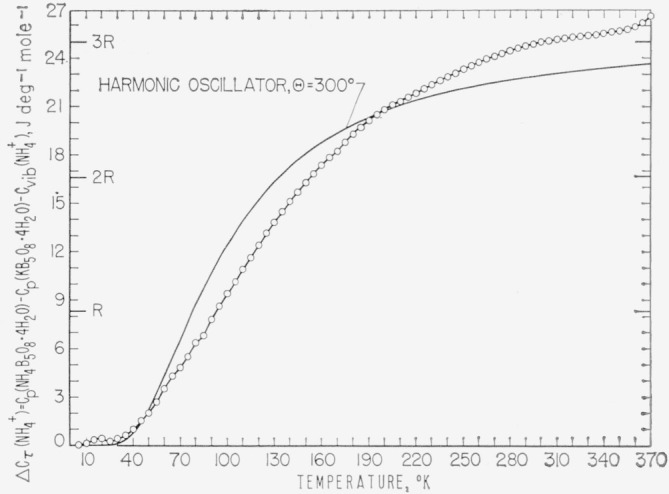
Heat capacity from the torsional or rotational motions of *NH_4_+* ion and the heat capacity of a harmonic oscillator.

A calculation similar to those presented earlier by Staveley et al. [15, 16, 17] was performed with the heat-capacity results obtained on APT and PPT. The results are shown in figure 5. APT and PPT are both orthorhombic, $\text{Aba}2-C_{2v}^{17}$, with crystal constants *a* = 11.324 Å, *b* = 11.029 Å, and *c* = 9.235 Å and *a* = 11.065 Å, *b* = 11.171 Å, and *c* = 9.054 Å, respectively [19]. The ionic radius of ammonium ion is 1.48 Å and that of the potassium ion is 1.33 Å [20]. The above crystal constants indicate that the specific volume of PPT is about 3 percent smaller than that of APT. The forces between the cation and anion are, therefore, expected to be somewhat different in the two salts, and the assumptions regarding the similarity in the contributions to the heat capacity other than from ∆*C_τ_*(NH_4_^+^) may not be completely valid. The internal and torsional motions of the anion and the water of hydration may be significantly different in the two salts. The rubidium ion with an ionic radius of 1.48 A [20] would be expected to form a salt with crystal constants close to those of the ammonium salt.

The results in the region of the upper temperature limit of measurements shown in figure 5 suggest that the ∆*C_τ_*(NH_4_^+^) in APT approximates the value 3*R* of a fully excited classical torsional oscillator. The results reported by Staveley et al. [15, 16, 17] on ammonium and rubidium salts of tetraphenylboron, stannic chloride, stannic bromide, and hexafluorophosphate are considerably below the 3*R* value. In the tetraphenylboron salt [17] the ∆*C_τ_*(NH_4_^+^) is shown to be about $\frac{5}{2}R$ at 300 °K, the upper limit of their measurements, and increasing. The ∆*C_τ_*(NH_4_^+^) of both ammonium stannic chloride and stannic bromide is shown to have a maximum followed by an asymptotic decrease with temperature [16] related to a hindered rotator behavior.

If heat-capacity measurements were made on rubidium pentaborate tetrahydrate (RPT) and the results used to calculate ∆*C_τ_*(NH_4_^+^) the values in the upper temperature region are expected to be higher than those shown in figure 5. The results of the heat-capacity measurements of Davies and Staveley [17] on ammonium, potassium, and rubidium salts of tetraphenylboron show that above 200 °K the heat capacity of the potassium salt is higher than that of the rubidium salt. The measurements of Morfee et al., [16] show also that the heat capacity of potassium stannic bromide is greater at the higher temperatures (above about 100 °K) than that of the corresponding rubidium salt. The considerably higher values than 3*R* expected for ∆*C_τ_*(NH_4_^+^), if the heat capacity of RPT were used instead, would indicate that the values close to 3*R* obtained for ∆*C_τ_*(NH_4_^+^) with APT and PPT measurements are fortuitous. For the simpler salts, for example the bromides [21], iodides [21], and acid fluorides [22, 23], the heat capacities of the rubidium salts are higher than those of the potassium salts. Therefore, it seems that the heat-capacity contributions from the various sources in complex salts, such as those of the pentaborate, are dependent in a complicated way on, among others, the cation present.

The ∆*C_τ_*(NH_4_^+^) obtained was compared with the heat capacity of a harmonic oscillator. Although the NH_4_^+^ ion in APT is in an asymmetric environment, the best average frequency was determined. In figure 5 the Einstein heat capacity with *θ* = 300 deg is compared with ∆*C_τ_*(NH_4_^+^). The values of ∆*C_τ_*(NH_4_^+^) differ by +100 percent at 10 °K and +8 percent at 300 °K. It is seen that ∆*C_τ_*(NH_4_^+^) behaves considerably different from the heat capacity of a simple torsional oscillator. An attempt was also made to fit the ∆*C_τ_*(NH_4_^+^) values obtained by Davies and Staveley [17] on ammonium tetraphenylboron, where the NH_4_^+^ ion is in a more symmetric environment, with the heat capacity of a harmonic oscillator. Although the agreement is better, the discrepancies indicate that the oscillation is not simple and that the heat-capacity contributions for the constituents of a system are affected in a complicated way by any substituent.

## Figures and Tables

**Table 1:** Chemical analysis of ammonium pentaborate tetrahydrate, *NH_4_B_5_O_8_·4H_2_O* Gram molecular weight = 272.150 g

	Percentage		
	PCBC^a^	Theoretical	This work
			
(NH_4_)_2_O	9.52	9.57	9.43
B_2_O_3_	63.84	63.96	63.59
H_2_O	…………	26.47	
Cl	0.000017		
SO_3_	.00006		
Fe	.00005		
Heavy metals as Pb	.00015		
As_2_O_3_ Less than..	.00002		
P_2_O_5_ do	.00005		
Mn do	.00001		
$\begin{array}{l} {\text{SiO}}_{2}\\ {\text{Al}}_{2}O_{3}\\ \text{CaO}\\ \text{MgO}\\ {\text{Na}}_{2}O \end{array}\}--------------\{\text{Less than sensitivity of Pacific Coast Borax Co}.,\;\text{methods}.$			
(NH_4_)_2_O:B_2_O_3_ Ratio	1:5.01	1:5	1:5.07

a Pacific Coast Borax Company.

**Table 2:** Chemical analysis of potassium pentaborate tetrahydrate, *KB_5_O_8_·4H_2_O* Gram molecular weight = 293.214 g

	Percentage			
	PCBC^a^	Theoretical	This work	
			Observed	Normalized
				
K_2_O	16.21	16.06	16.07	16.09
B_2_O_3_	59.31	59.37	59.18	59.25
H_2_O	…………	24.57	24.63	24.66
Na_2_O	none			
Cl	0.008			
SO_3_	.0002			
SiO_2_	.0038			
Al_2_O_3_	.0051			
F_2_O_3_	.0003			
CaO	.0012			
MgO Less than—	.0005			
As_2_O_3_	.0007			
P_2_O_5_	.0009			
Pb	.0050			
Mn Less than—	.00001			
K_2_O:B_2_O_3_ Ratio	1:4.95	1:5	4:4.98	
		
Total of K_2_O, B_2_O_3_, and H_2_O			99.88	100.00

a Pacific Coast Borax Company.

**Table 3:** Chemical analysis of sodium pentaborate pentahydrate, *NaB_5_O_8_·5H_2_O* Gram molecular weight = 295.117 g

	Percentage			
	PCBC^a^	Theoretical	This work	
			Observed	Normalized
				
Na_2_O	10.59	10.50	10.64	10.62
B_2_O_3_	58.64	58.98	58. 54	58. 45
H_2_O	…………	30.52	30.98	30. 93
Cl	0.0046			
SO_3_	.0014			
SiO_2_	.0014			
Al_2_O_3_	.0047			
Fe_2_O_3_	.0035			
CaO	.0080			
MgO	.0005			
P_2_O_5_	.0041			
As_2_O_3_	.0001			
Pb	.0015			
Mn	.00025			
Na_2_O:B_2_O_3_ Ratio	1:4.93	1:5	1:4.90	
		
Total of Na_2_O, B_2_O_3_, and H_2_O			100.16	100.00

a Pacific Coast Borax Company.

**Table 4:** Percentages of the suspected impurities based on the analyses on alkali oxide, boric oxide and water contents

Compound	Impurity	Method of Analysis		
		M_2_O	B_2_O_3_	H_2_O
				
APT	B(OH)_3_	1.4	4.8	
PPT	B(OH)_3_	−0.2	2.6	0.4
SPP	Na_2_B_4_O_7_·10H_2_O	2.1	2.4	2.5

**Table 5:** Observed heat capacities of ammonium pentaborate tetrahydrate (*NH_4_B_5_O_8_·4H_2_O*) Gram molecular weight = 272.150 g, *T* deg K*=t* deg C + 273.15

Run No.	*T* a	*C_P_*^b^
	*°K*	*J deg*^−1^ *mole*^−1^
1	c 52.3798	82.092
	55.4564	87.804
	59.8250	95.826
	64.6501	104.25
	68.9856	111.18
	73.0430	117.29
	77.3063	123.76
	81.7702	130.41
2	11.4684	4.268
	12.2470	5.041
	12.9902	5.857
	13.7754	6.831
	14.6079	7.881
	15.5078	9.139
	16.4598	10.516
	17.5058	12.118
	18.7824	14.174
	20.1809	16.554
	21.5636	19.032
	23.0268	21.736
	24.5876	24.749
	26.2042	29.964
	27.9198	31.402
	29.9899	35.709
	32.1900	40.385
	34.2854	43.807
	36.6468	49.984
	39.7470	56.529
	43.5102	64.396
	47.0718	71.681
	50.7106	78.998
	53.3932	84.159
	56.3964	89.765
	60.7088	97.628
3	79.7048	126.60
	83.7367	132.96
	87.8826	138.91
	91.8441	144.06
	95.6642	148.85
	99.3594	153.49
	102.9710	157.99
	106.5092	162.33
	110.2500	167.37
	114.1990	171.64
	118.1925	176.34
	122.2382	181.13
	126.1821	185.61
4	154.0512	216.64
	158.8058	221.58
	163.5319	226.65
	168.2332	231.54
	172.8415	236.31
	177.5292	241.08
	182.2946	246.17
	186.9958	250.78
	191.6386	255.56
5	185.3702	248.91
	188.8388	252.08
	193.3818	257.29
	198.9176	262.78
	205.4147	269.22
	211.7558	275.38
	218.4874	282.04
	225.5958	289.18
	232.5298	295.51
	239.3223	302.38
	246.0784	308.92
	253.7222	316.48
	260.8091	322.92
	267.2680	328.97
	273.6075	335.09
	279.8340	340.69
6	277.7998	338.07
	285.7512	345.54
	293.5821	352.74
	301.2508	359.45
	308.7752	366.45
	316.1723	373.00
	323.4430	379.46
	330.5918	385.73
	337.6318	391.78
	344.9387	397.97
	352.4211	404.64
	359.6954	410.85
	366.8040	417.64
7	127.8625	187.44
	134.7264	195.30
	141.6874	203.00
	147.7845	209.64
	153.2160	215.54
	158.4969	221.19

a *T* is the mean temperature of the heating interval.

b *C_P_* is the observed mean heat capacity over the interval.

c The temperatures given are believed to be accurate to 0.01 °K. The figures beyond the second decimal are significant only insofar as small temperature differences are concerned.

**Table 6:** Molal thermal functions for ammonium pentaborate tetrahydrate (*NH_4_B_5_O_8_·4H_2_O*) Gram molecular weight=272.150 g, *T* deg K*=t* deg C + 273.15

*T*	*C_P_*	$\left(H_{T}-H_{0}^{C}\right)$	$\frac{\left(H_{T}-H_{0}^{C}\right)}{T}$	*S_T_*	$-\left(G_{T}-H_{0}^{C}\right)$	$\frac{-\left(G_{T}-H_{0}^{C}\right)}{T}$
						
*°K*	*J*/*deg*	*J*	*J*/*deg*	*J*/*deg*	*J*	*J*/*deg*
0.00	0.000	0.000	0.000	0.000	0.000	0.000
5.00	0.327	0.405	0.081	0.107	0.131	0.026
10.00	2.607	6.532	0.653	0.870	2.165	0.216
15.00	8.224	32.215	2.148	2.876	10.924	0.728
20.00	16.386	93.166	4.658	6.326	33.363	1.668
25.00	25.512	197.30	7.892	10.936	76.105	3.044
30.00	35.709	350.05	11.668	16.478	144.29	4.810
35.00	46.371	555.11	15.860	22.779	242.15	6.918
40.00	57.033	813.76	20.344	29.670	373.06	9.326
45.00	67.437	1125.0	25.001	36.991	539.56	11.990
50.00	77.514	1487.6	29.752	44.621	743.49	14.870
55.00	87.128	1899.4	34.535	52.464	986.13	17.930
60.00	96.263	2358.1	39.301	60.440	1268.3	21.139
65.00	105.01	2861.5	44.024	68.495	1590.7	24.472
70.00	112.74	3406.2	48.660	76.564	1953.3	27.905
75.00	120.20	3988.6	53.181	84.598	2356.2	31.417
80.00	127.77	4608.6	57.608	92.598	2799.2	34.991
85.00	134.71	5265.0	61.941	100.55	3282.1	38.613
90.00	141.61	5955.9	66.176	108.45	3804.7	42.274
95.00	148.07	6680.2	70.318	116.28	4366.5	45.964
100.00	154.32	7436.3	74.363	124.04	4967.4	49.674
105.00	160.46	8223.3	78.317	131.71	5606.8	53.398
110.00	166.50	9040.7	82.188	139.32	6284.4	57.131
115.00	172.42	9888.1	85.983	146.85	6999.9	60.868
120.00	178.26	10765	89.707	154.31	7752.8	64.607
125.00	184.04	11671	93.365	161.71	8542.9	68.343
130.00	189.76	12605	96.962	169.04	9369.8	72.075
135.00	195.42	13568	100.50	176.31	10233	75.801
140.00	201.01	14559	103.99	183.51	11133	79.519
145.00	206.55	15578	107.44	190.66	12068	83.229
150.00	212.02	16625	110.83	197.76	13039	86.928
155.00	217.44	17698	114.18	204.80	14046	90.617
160.00	222.81	18799	117.49	211.79	15087	94.295
165.00	228.13	19926	120.77	218.73	16163	97.960
170.00	233.26	21080	124.00	225.61	17274	101.61
175.00	238.60	22259	127.20	232.45	18420	105.25
180.00	243.76	23465	130.36	239.25	19599	108.88
185.00	248.86	24697	133.50	245.99	20812	112.50
190.00	253.92	25954	136.60	252.70	22059	116.10
195.00	258.92	27236	139.67	259.36	23339	119.69
200.00	263.87	28543	142.71	265.98	24652	123.26
205.00	268.79	29875	145.73	272.55	25998	126.82
210.00	273.65	31231	148.72	279.09	27378	130.37
215.00	278.55	32611	151.68	285.58	28789	133.90
220.00	283.44	34016	154.62	292.04	30233	137.42
225.00	288.32	25446	157.54	298.47	31710	140.93
230.00	293.20	36899	160.43	304.86	33218	144.43
235.00	298.04	38377	163.31	311.22	34758	147.91
240.00	302.87	39880	166.17	317.54	36330	151.38
245.00	307.68	41406	169.00	323.84	37934	154.83
250.00	312.46	42956	171.83	330.10	39568	158.27
255.00	317.21	44531	174.63	336.33	41234	161.70
260.00	321.93	46129	177.42	342.54	42932	165.12
265.00	326.63	47750	180.19	348.72	44660	168.53
270.00	331.29	49395	182.94	354.86	46419	171.92
273.15	334.22	50443	184.67	358.72	47543	174.05
275.00	335.93	51063	185.68	360.99	48208	175.30
280.00	340.54	52754	188.41	367.08	50029	178.67
285.00	345.13	54468	191.12	373.15	51879	182.03
290.00	349.68	56205	193.81	379.19	53760	185.38
295.00	354.22	57965	196.49	385.21	55671	188.72
298.15	357.06	59085	198.17	388.98	56890	190.81
300.00	358.72	59747	199.16	391.20	57612	192.04
305.00	363.20	61552	201.81	397.16	59583	195.35
310.00	367.65	63379	204.45	403.11	61584	198.66
315.00	372.08	65229	207.08	409.02	63614	201.95
320.00	376.49	67100	209.69	414.92	65674	205.23
325.00	380.87	68993	212.29	420.79	67763	208.50
330.00	385.22	70909	214.87	426.64	69882	211.76
335.00	389.57	72846	217.45	432.46	72029	215.01
340.00	393.90	74804	220.01	438.27	74206	218.25
345.00	398.21	76785	222.56	444.05	76412	221.48
350.00	402.53	78786	225.10	449.81	78647	224.71
355.00	406.85	80810	227.63	455.55	80910	227.92
360.00	411.20	82855	230.15	461.27	83202	231.12
365.00	415.64	84922	232.66	466.97	85523	234.31
370.00	420.19	87012	235.17	472.66	87872	237.49
373.15	423.14	88340	236.74	476.23	89366	239.49

$H_{0}^{C}$ apply to the reference state of the solid at 0 °K.

**Table 7:** Observed heat capacities of potassium pentaborate tetrahydrate (*KB_5_O_8_·4H_2_O*) Gram molecular weight=293.214 g, *T* deg *K=t* deg C + 273.15

Run No.	*T*^a^	*C_P_*^b^
	*°K*	*J deg*^−1^ *mole*^−1^
1	c 82.3034	124.47
	84.5045	127.41
	87.2906	130.73
	90.4015	134.47
	94.2352	138.65
	97.7969	142.38
	100.8126	145.88
	104.4460	149.72
	109.2458	154.79
	114.4401	160.14
	119.4561	165.24
	124.3238	169.94
2		
	122.5274	168.51
	126.7802	172.44
	132.0640	177.73
	137.7528	183.65
	143.2724	189.10
	148.7043	194.28
	154.0496	199.69
	159.4711	205.37
	165.1884	210.55
	171.1866	215.30
	177.2576	222.28
	183.3799	227.74
	189.7612	233.17
	196.3811	239.71
	203.0422	245.48
	209.5620	251.83
	215.9440	258.02
3		
	17.6472	11.985
	19.7382	15.512
	22.2888	20.078
	25.1550	25.547
	27.8585	30.962
	30.2911	35.903
	32.7529	41.031
	35.3106	46.382
	37.6918	51.289
	40.0046	56.023
	42.2754	60.535
	44.8340	65.552
	47.8084	71.345
	50.9450	77.261
	54.4675	83.506
	58.0818	89.653
	61.7500	95.681
	65.5484	101.56
4		
	57.0740	87.903
	60.5248	93.626
	64.0627	99.322
	67.7316	104.69
	71.2346	109.58
	75.3972	115.25
	80.1645	121.60
	84.6696	127.49
	88.9634	132.78
5		
	197.9566	240.80
	205.1094	247.60
	212.5784	254.49
	220.3804	261.68
	228.0024	268.67
	235.4532	275.46
	242.7348	282.04
	249.6717	288.22
	257.3066	295.06
	265.1866	302.01
	273.2946	309.20
	281.2286	315.99
	289.0028	323.08
	296.6320	329.04
	305.3462	336.59
	315.1084	344.83
	324.6656	352.76
6		
	333.5876	360.24
	342.4691	367.37
	351.1885	374.38
	359.7526	381.26
	368.1638	388.43

a *T* is the mean temperature of the heating interval.

b *C_P_* is the observed mean heat capacity over the interval.

c The temperatures given are believed to be accurate to 0.01 °K. The figures beyond the second decimal are significant only insofar as small temperature differences are concerned.

**Table 8:** Molal thermal functions for potassium pentaborate tetrahydrate (*KB_5_O_8_·4H_2_O*) Gram molecular weight=293.214 g, *T* deg K=*t* deg C + 273.15

*T*	*C_P_*	$\left(H_{T}-H_{O}^{C}\right)$	$\frac{\left(H_{T}-H_{O}^{C}\right)}{T}$	*S_T_*	$-\left(G_{T}-H_{O}^{C}\right)$	$\frac{-\left(G_{T}-H_{O}^{C}\right)}{T}$
						
*°K*	*J*/*deg*	*J*	*J*/*deg*	*J*/*deg*	*J*	*J*/*deg*
0.00	0.000	0.000	0.000	0.000	0.000	0.000
5.00	.308	.385	.077	.103	.128	.026
10.00	2.460	6.163	.616	.822	2.055	.205
15.00	7.871	30.557	2.037	2.727	10.341	.689
20.00	15.959	89.428	4.471	6.058	31.730	1.586
25.00	25.240	191.96	7.678	10.595	72.921	2.917
30.00	35.285	343.14	11.438	16.080	139.26	4.642
35.00	45.729	545.59	15.588	22.301	234.94	6.713
40.00	55.941	799.91	19.998	29.078	363.20	9.080
45.00	65.891	1104.6	24.548	36.245	526.37	11.697
50.00	75.439	1458.2	29.164	43.686	726.10	14.522
55.00	84.423	1858.2	33.785	51.303	963.53	17.519
60.00	92.753	2301.3	38.355	59.010	1239.3	20.655
65.00	100.72	2785.2	42.850	66.752	1553.7	23.903
70.00	107.85	3306.9	47.241	74.481	1906.8	27.240
75.00	114.70	3863.4	51.512	82.157	2298.4	30.645
80.00	121.37	4453.6	55.670	89.773	2728.2	34.103
85.00	127.90	5077.0	59.729	97.329	3196.0	37.600
90.00	133.79	5731.4	63.682	104.81	3701.4	41.127
95.00	139.39	6414.5	67.521	112.19	4244.0	44.673
100.00	144.88	7125.2	71.252	119.48	4823.2	48.232
105.00	150.27	7863.1	74.887	126.68	5438.6	51.797
110.00	155.56	8627.7	78.434	133.80	6089.9	55.363
115.00	160.75	9418.5	81.900	140.83	6776.5	58.926
120.00	165.83	10235	85.292	147.78	7498.0	62.483
125.00	170.88	11077	88.615	154.65	8254.1	66.033
130.00	175.89	11944	91.875	161.45	9044.4	69.572
135.00	180.88	12836	95.080	168.18	9868.5	73.100
140.00	185.84	13753	98.232	174.85	10726	76.615
145.00	190.78	14694	101.34	181.45	11617	80.116
150.00	195.68	15660	104.40	188.01	12541	83.603
155.00	200.55	16651	107.43	194.50	13497	87.076
160.00	205.39	17666	110.41	200.95	14485	90.534
165.00	210.19	18705	113.36	207.34	15506	93.977
170.00	214.95	19768	116.28	213.68	16559	97.404
175.00	219.70	20854	119.17	219.98	17643	100.82
180.00	224.38	21964	122.02	226.24	18759	104.21
185.00	229.05	23098	124.85	232.45	19905	107.60
190.00	233.68	24255	127.66	238.62	21083	10096
195.00	238.30	25435	130.44	244.75	22291	114.31
200.00	242.90	26638	133.19	250.84	23530	117.65
205.00	247.51	27864	135.92	256.90	24800	120.97
210.00	252.13	29113	138.63	262.92	26099	124.28
215.00	256.74	30385	141.33	268.90	27429	127.58
220.00	261.34	31680	144.00	274.86	28788	130.86
225.00	265.92	32999	146.66	280.78	30177	134.12
230.00	270.49	34340	149.30	286.68	31596	137.37
235.00	275.03	35703	151.93	292.54	33044	140.61
240.00	279.55	37090	154.54	298.38	34521	143.84
245.00	284.05	38499	157.14	304.19	36028	147.05
250.00	288.53	39930	159.72	309.97	37563	150.25
255.00	292.98	41384	162.29	315.73	39128	153.44
260.00	297.41	42860	164.85	321.46	40720	156.62
265.00	301.82	44358	167.39	327.17	42342	159.78
270.00	306.21	45878	169.92	332.85	43992	162.93
273.15	308.96	46847	171.51	336.42	45046	164.91
275.00	310.57	47420	172.44	338.51	45671	166.07
280.00	314.91	48984	174.94	344.15	47377	169.20
285.00	319.23	50569	177.44	349.76	49112	172.32
290.00	323.52	52176	179.92	355.35	50875	175.43
295.00	327.79	53804	182.39	360.91	52665	178.53
298.15	330.48	54841	183.94	364.41	53808	180.47
300.00	332.05	55454	184.85	366.46	54484	181.61
305.00	336.29	57125	187.29	371.98	56330	184.6
310.00	340.51	58817	189.73	377.49	58204	187.75
315.00	344.70	60530	192.16	382.97	60105	190.81
320.00	348.88	62264	194.57	388.43	62033	193.85
325.00	353.03	64019	196.98	393.87	63989	196.89
330.00	357.16	65794	199.38	399.29	65972	199.92
335.00	361.26	67590	201.76	404.69	67982	202.93
340.00	365.35	69407	204.14	410.07	70019	205.94
345.00	369.41	71243	206.50	415.44	72083	208.94
350.00	373.45	73101	208.86	420.78	74173	211.92
355.00	377.46	74978	211.21	426.11	76290	214.90
360.00	381.46	76875	213.54	431.42	78434	217.87
365.00	385.42	78792	215.87	436.70	80605	220.83
370.00	389.37	80729	218.19	441.97	82801	223.79
373.15	391.83	81960	219.64	445.29	84199	225.64

$H_{0}^{C}$ apply to the reference state of the solid at 0 °K.

**Table 9:** Observed heat capacities of sodium pentaborate pentahydrate (*NaB_5_O_8_·5H_2_O*) Gram molecular weight=295.117 g, *T* deg K*=t* deg C + 273.15

Run No.	*T* a	*C_P_* b
1	*°K*	*J deg*^−1^ *mole*^−1^
	c 83.4065	124.37
	88.6085	132.06
	92.7627	137.50
	97.3204	143.41
	101.6895	149.06
	106.2244	154.84
	110.9289	160.82
	115.4754	166.51
	119.8804	172.15
	124.1608	177.35
	128.3349	182.27
	132.8928	187.80
	137.8308	193.69
	142.8158	199.58
	148.3402	206.04
	154.2458	212.94
	160.0278	219.60
	165.6592	226.10
2		
	164.1770	224.39
	169.7858	230.82
	175.2371	237.01
	180.6933	242.88
	186.1680	247.49
	192.8228	255.46
	200.6042	263.77
	208.1534	274.86
	214.8802	283.31
	220.8498	290.09
	226.7036	296.50
	232.5463	303.08
	238.3790	309.68
	243.8250	315.73
	249.1776	321.57
	254.9914	327.98
	260.9762	334.45
3		
	247.7110	319.95
	252.7798	325.54
	258.7220	331.96
	264.6066	338.25
	270.4336	344.61
	276.3932	350.88
	282.4880	357.31
	288.4866	363.57
4		
	15.8520	9.200
	17.3346	11.104
	18.8288	13.302
	20.2713	15.540
	21.8516	18.086
	23.5410	20.907
	25.4136	24.192
	27.3312	27.690
	29.4477	31.546
	32.0088	36.349
	34.8345	41.750
	38.0104	47.805
	41.5702	54.494
	45.7972	62.346
	50.5750	71.141
	55.3838	79.630
	60.4812	88.489
	66.0802	97.873
	72.4908	107.81
5		
	58.5476	85.150
	63.1808	93.159
	68.3146	101.42
	74.2438	110.53
	80.4764	119.97
	85.5548	127.70
	90.3071	134.32
	95.4888	141.09
6		
	153.3534	211.82
	159.1856	218.60
	164.8744	225.19
	170.4274	231.53
7		
	161.3418	220.79
	167.1078	227.40
	172.5546	233.81
	177.9436	239.66
	183.2148	245.70
	188.3760	251.54
	193.4302	258.34
	198.9778	265.70
	205.5902	273.10
	211.4822	279.82
	216.0996	284.99
8		
	278.6015	353.16
	284.7018	359.50
	290.6984	365.78
	296.6560	371.93
	302.6415	378.23
	308.5999	384.49
	312.8014	388.84
	318.6032	394.92
	324.3276	401.10
	330.4356	408.02
	337.3630	416.08
	344.9336	444.15
9		
	268.9421	342.92
	270.9374	345.18
	272.9196	347.45
	274.8890	349.51
	276.8471	351.36
	278.7942	353.50
10		
	183.5999	246.78
	202.5076	269.22
	221.3134	290.69

a *T* is the mean temperature of the heating interval.

b *C_P_* is the observed mean heat capacity over the interval.

c The temperatures given are believed to be accurate to 0.01° K. The figures beyond the second decimal are significant only insofar as small temperature differences are concerned.

**Table 10:** Molal thermal functions for sodium pentaborate pentahydrate (*NAB_5_O_8_·5H_2_O*) Gram molecular weight=295.117 g, *T* deg K=*t* deg C + 273.15

*T*	*C_P_*	$\left(H_{T}-H_{0}^{C}\right)$	$\frac{\left(H_{T}-H_{0}^{C}\right)}{T}$	*S_T_*	$-\left(G_{T}-H_{0}^{C}\right)$	$\frac{-\left(G_{T}-H_{0}^{C}\right)}{T}$
						
*°K*	*J*/*deg*	*J*	*J*/*deg*	*J*/*deg*	*J*	*J*/*deg*
0.00	0.000	0.000	0.000	0.000	0.000	0.000
5.00	.303	.378	.076	.101	.126	.025
10.00	2.412	6.046	.605	.806	2.017	.202
15.00	7.658	29.873	1.992	2.667	10.136	.676
20.00	15.102	86.499	4.325	5.874	30.976	1.549
25.00	23.425	182.40	7.296	10.119	70.581	2.823
30.00	32.552	322.21	10.740	15.192	133.55	4.452
35.00	42.084	508.60	14.531	20.920	223.59	6.388
40.00	51.526	742.67	18.567	27.157	343.60	8.590
45.00	60.857	1023.7	22.749	33.766	495.77	11.017
50.00	70.088	1351.2	27.023	40.658	681.73	13.635
55.00	78.950	1723.8	31.342	47.755	902.69	16.413
60.00	87.643	2140.4	35.673	54.998	1159.5	19.325
65.00	96.143	2600.0	40.000	62.352	1452.9	22.352
70.00	104.00	3100.6	44.294	69.767	1783.1	25.473
75.00	111.63	3639.7	48.529	77.203	2150.6	28.674
80.00	119.24	4216.9	52.711	84.651	2555.2	31.940
85.00	126.87	4832.3	56.850	92.110	2997.1	35.260
90.00	133.89	5484.4	60.938	99.563	3476.3	38.625
95.00	140.41	6170.3	64.950	106.98	3992.7	42.028
100.00	146.88	6888.5	68.885	114.35	4546.0	45.460
105.00	153.30	7639.0	72.752	121.67	5136.0	48.915
110.00	159.64	8421.3	76.558	128.94	5762.6	52.387
115.00	165.94	9235.3	80.307	136.18	6425.4	55.873
120.00	172.11	10080	84.004	143.37	7124.3	59.369
125.00	178.23	10956	87.651	150.52	7859.1	62.873
130.00	184.30	11863	91.252	157.63	8629.5	66.381
135.00	190.29	12799	94.809	164.70	9435.3	69.891
140.00	196.23	13766	98.326	171.73	10276	73.403
145.00	202.11	14761	101.80	178.72	11153	76.914
150.00	207.96	15787	105.24	185.67	12064	80.424
155.00	213.76	16841	108.65	192.58	13009	83.930
160.00	219.51	17924	112.03	199.46	13989	87.433
165.00	225.18	19036	115.37	206.30	15004	90.932
170.00	230.83	20176	118.68	213.11	16052	94.425
175.00	236.48	21344	121.97	219.88	17135	97.913
180.00	242.12	22541	125.23	226.62	18251	101.39
185.00	247.74	23765	128.46	233.33	19401	104.87
190.00	253.56	25018	131.68	240.01	20594	108.34
195.00	259.78	26302	134.88	246.68	21801	111.80
200.00	266.07	27616	138.08	253.34	23051	115.26
205.00	272.33	28962	141.28	259.98	24334	118.70
210.00	278.15	30339	144.47	266.62	25651	122.15
215.00	283.81	31744	147.65	273.23	27000	125.58
220.00	289.39	33177	150.80	279.82	28383	129.01
225.00	294.98	34638	153.95	286.38	29799	132.44
230.00	300.53	36126	157.07	292.93	31247	135.86
235.00	306.06	37643	160.18	299.45	32728	139.27
240.00	311.56	39187	163.28	305.95	34241	142.67
245.00	317.03	40759	166.36	312.43	35787	146.07
250.00	322.48	42357	169.43	318.89	37366	149.46
255.00	327.90	43983	172.48	325.33	38976	152.85
260.00	333.30	45636	17552	331.75	40619	156.23
265.00	338.66	47316	178.55	338.15	42294	159.60
270.00	344.01	49023	181.57	344.53	44000	162.96
273.15	347.36	50112	183.46	348.54	45092	165.08
275.00	349.33	50756	184.57	350.89	45739	166.32
280.00	354.62	52516	187.56	357.23	47509	169.68
285.00	359.87	54302	190.53	363.56	49311	173.02
290.00	365.14	56115	193.50	369.86	51145	176.36
295.00	370.28	57953	196.45	376.15	53010	179.69
298.15	373.55	59125	198.31	380.10	54201	181.79
300.00	375.47	59818	199.39	382.41	54906	183.02
305.00	380.68	61708	202.32	388.66	56834	186.34
310.00	385.91	63625	205.24	394.90	58793	189.65
315.00	391.17	65567	208.15	401.11	60783	192.96
320.00	396.50	67536	211.05	407.31	62804	196.26
325.00	401.96	69533	213.95	413.50	64856	199.56
330.00	407.52	71556	216.84	419.68	66939	202.85
335.00	413.24	73608	219.73	425.85	69053	206.13
340.00	419.05	75689	222.61	432.02	71197	209.40
345.00	424.75	77798	225.50	438.18	73373	212.68

$H_{0}^{C}$ apply to the reference state of the solid at 0 °K.

## Footnotes

1 Figures in brackets indicate the literature references at the end of this paper.
